# Evolutionary optimization of classifiers and features for single-trial EEG Discrimination

**DOI:** 10.1186/1475-925X-6-32

**Published:** 2007-08-23

**Authors:** Malin CB Åberg, Johan Wessberg

**Affiliations:** 1 Department of Neuroscience and Physiology, Göteborg University, Göteborg, 413 90, Sweden

## Abstract

**Background:**

State-of-the-art signal processing methods are known to detect information in single-trial event-related EEG data, a crucial aspect in development of real-time applications such as brain computer interfaces. This paper investigates one such novel approach, evaluating how individual classifier and feature subset tailoring affects classification of single-trial EEG finger movements. The discrete wavelet transform was used to extract signal features that were classified using linear regression and non-linear neural network models, which were trained and architecturally optimized with evolutionary algorithms. The input feature subsets were also allowed to evolve, thus performing feature selection in a wrapper fashion. Filter approaches were implemented as well by limiting the degree of optimization.

**Results:**

Using only 10 features and 100 patterns, the non-linear wrapper approach achieved the highest validation classification accuracy (subject mean 75%), closely followed by the linear wrapper method (73.5%). The optimal features differed much between subjects, yet some physiologically plausible patterns were observed.

**Conclusion:**

High degrees of classifier parameter, structure and feature subset tailoring on individual levels substantially increase single-trial EEG classification rates, an important consideration in areas where highly accurate detection rates are essential. Also, the presented method provides insight into the spatial characteristics of finger movement EEG patterns.

## Background

Electroencephalography (EEG) is a long-established method for investigation of event-related cortical processing, where the electrical activity of the brain is recorded in high-resolution real time by scalp electrodes. The resulting collection of signals is highly complex, being multivariate, non-stationary, extremely noisy and high-dimensional [1]. These inherent properties result in analysis difficulties traditionally overcome by offline averaging of numerous events, time-fixed to a stimulus. In contrast, machine learning approaches provide tools for detection and classification of cortical patterns in real time. Moreover, these methods are valuable for efficient data dimensionality reduction and feature selection, issues which receive growing attention in neuroscience as hardware technology, particularly multi-channel EEG and fMRI, offers increasingly improved spatial and temporal resolution.

The real-time pattern identification potential is particularly important for applications such as brain computer interfaces (BCI) – devices allowing the brain to directly control external appliances through the detection of given cortical patterns. Studies in this area have shown that it is, for example, possible to distinctly differentiate between the single-trial EEG patterns produced during right and left finger movement, both actual and imagined, in sedentary subjects [2,3].

Much motor-based BCI-research has focused exclusively on the primary motor cortex, restricting signal registration to a few predefined, mainly central, electrode locations [4-8]. However, motor actions generate relevant EEG activity in other complementary areas as well [9-11]. Aspects that vary not only throughout movement but also between individuals, such as dipole orientation, affect the spatial EEG pattern and make it difficult to predict which electrodes provide relevant information without imposing potentially restricting assumptions about the signal source. Similarly, BCIs typically limit EEG signal characterization to preset frequency ranges. Studies focusing on optimizing individual feature sets have, however, reported that between subjects, areas and frequencies most relevant for laterality discrimination vary widely [10-12]. As pointed out by Graimann et al [11], a BCI based on only one phenomenon, such as the event-related potential or event-related synchronisation and desynchronisation, will be less robust and accurate than a BCI based on both or more.

Including all possible signal features would, however, result in an extremely high dimensional feature space, given the myriad of methods for transforming and describing the EEG signal mathematically. As a consequence of the curse of dimensionality [13], the number of observations must be drastically increased as the feature space grows in order to maintain the same classification results. The extent of the acquired EEG signals is for practical reasons limited and thus the number of features used must be minimized.

Common methods of dimensionality reduction include principal component analysis (PCA) and linear discriminant analysis (LDA) where the original features are mathematically projected onto a lower-dimensional space. Here, however, we look at dimensionality reduction from a combinatorial perspective and attempt to detect which combination of a limited number of features carry relevant information. This process, referred to as feature subset selection, involves discarding redundant or irrelevant features while promoting ones that maintain or improve classification accuracy [10,14]. An optimized feature set leads to faster, computationally more efficient and, most importantly, more accurate classification. Also, a properly designed feature selection process generates a feature relevance ranking, describing how well signal components capture elements of the cortical processing related to given stimuli. There are two distinct approaches to feature subset optimization, termed wrapper and filter feature selection [15]. The former involves simultaneous and continuous optimization of classifier parameters and feature subset. The filter method, on the other hand, involves feature subset selection independent of classifier parameter optimization. The wrapper approach typically gives better results due to maximal integration between classifier and feature subset, yet filter feature subset selection is sometimes preferred since it usually requires less computer resources.

The combinatorial aspect of feature selection has been successfully explored by evolutionary algorithms (EA), within BCI research [11,12], and other areas [16,17], although not in combination with classifier tailoring. EAs are population-based optimization methods inspired by Darwinian evolution, which can, by proper parameter coding, optimize classifier and feature subsets by either the wrapper or filter approach. EAs are also suitable for optimizing classifier parameters, such as multilayer artificial neural network (ANN) weights and architecture [18,19]. ANNs can, given proper design and training, solve any classification problem and have proven effective at generalizing to unseen data [20]. However, standard ANN design procedures require complete external specification of the network architecture, typically based on time-consuming empirical exploration or crude system assumptions. In contrast, network optimization using EAs allows the architecture to evolve much like in biological systems, rendering user intervention or system postulations dispensable. Moreover, allowing evolution of not only internal architecture, but also the included features directly performs feature subset selection in a wrapper fashion. Other classification schemes, such as multiple linear regression (MLR), can be similarly optimized [21].

There is reason to believe that systematically tailoring classifiers and feature subsets for every individual will maximize extraction of relevant information, as opposed to noise, from the EEG. Consequently, the aim of this study was to design and compare methods for automatic classifier tailoring and feature subset optimization in order to maximize EEG pattern detection accuracy. The results have in part been previously presented in poster format [22].

## Methods

### EEG Acquisition and Pre-Processing

The study was performed in accordance with the Declaration of Helsinki and approved by the Göteborg University ethics committee. Four healthy untrained subjects, three female and one male, aged 24–43 years, one left-handed, participated in the study. The subjects, comfortably seated in a chair, were instructed to move either the left or the right index finger in a brisk, self-paced manner according to cues presented on a screen. The interval between the randomized cues was four seconds. Each cue was presented for three seconds, during which the subject moved the finger at a self-determined point. Between 250–900 movements were registered for each subject. Movements were recorded with accelerometers attached to the fingers (EGAX-5 monoaxial, Entran Inc., Fairfield, NJ, USA). EEG was acquired at a sampling rate of 256 Hz using active electrodes and the Active Two digital EEG amplifier and recording system from Biosemi, Inc. (Amsterdam, The Netherlands), with 32 scalp electrodes positioned according to the extended 10/20 system.

After amplification, the acquired data was high-pass filtered with cutoff frequency of 1 Hz and a reference average of all channels was subtracted. No notch filter was used. Epochs of -1000 to +500 ms relative to movement were extracted and visually inspected for eye blink artifacts. All data processing was performed with Matlab™(The Mathworks, Massachusetts, USA) software. In order to limit computing times, only 100 movements, randomly selected, were retained per subject. The epochs were divided into training (80%) and validation (20%) data sets containing equal numbers of left and right finger movements.

### Feature Extraction using Wavelets

The wavelet transform has been shown to be more effective in single-trial EEG characterization than traditional processing approaches [23]. The continuous wavelet transform (CWT) treats a function of time in constituent oscillations, localized in both time and frequency [24]. The CWT is defined as follows:

γ(s,τ)=∫f(t)Ψs,τ∗(t)dt
 MathType@MTEF@5@5@+=feaafiart1ev1aaatCvAUfKttLearuWrP9MDH5MBPbIqV92AaeXatLxBI9gBaebbnrfifHhDYfgasaacH8akY=wiFfYdH8Gipec8Eeeu0xXdbba9frFj0=OqFfea0dXdd9vqai=hGuQ8kuc9pgc9s8qqaq=dirpe0xb9q8qiLsFr0=vr0=vr0dc8meaabaqaciaacaGaaeqabaqabeGadaaakeaaiiGacqWFZoWzcqGGOaakcqWGZbWCcqGGSaalcqWFepaDcqGGPaqkcqGH9aqpdaWdbaqaaiabdAgaMjabcIcaOiabdsha0jabcMcaPiabfI6aznaaDaaaleaacqWGZbWCcqGGSaalcqWFepaDaeaacqGHxiIkaaGccqGGOaakcqWG0baDcqGGPaqkcqWGKbazcqWG0baDaSqabeqaniabgUIiYdaaaa@483D@

Ψs,τ(t)=1sΨ(t−τs)
 MathType@MTEF@5@5@+=feaafiart1ev1aaatCvAUfKttLearuWrP9MDH5MBPbIqV92AaeXatLxBI9gBaebbnrfifHhDYfgasaacH8akY=wiFfYdH8Gipec8Eeeu0xXdbba9frFj0=OqFfea0dXdd9vqai=hGuQ8kuc9pgc9s8qqaq=dirpe0xb9q8qiLsFr0=vr0=vr0dc8meaabaqaciaacaGaaeqabaqabeGadaaakeaacqqHOoqwdaWgaaWcbaGaem4CamNaeiilaWccciGae8hXdqhabeaakiabcIcaOiabdsha0jabcMcaPiabg2da9maalaaabaGaeGymaedabaWaaOaaaeaacqWGZbWCaSqabaaaaOGaeuiQdK1aaeWaaeaadaWcaaqaaiabdsha0jabgkHiTiab=r8a0bqaaiabdohaZbaaaiaawIcacaGLPaaaaaa@41FE@

where * denotes complex conjugation, *τ *is referred to as the translation, giving the position in time, and *s *the scale parameter, which is inversely related to the frequency content. Ψ(*τ*) is called the mother wavelet, and in this study the standard Daubechies function is used. The discrete wavelet transform (DWT), in turn, is the result of selecting scales and translations based on powers of two, yielding a more efficient yet as accurate analysis.

In order to limit the analysis to a smaller number of discriminative signal features, the DWT was applied to the difference of the average of the right and left finger movement epochs of the training data set, and the five coefficients accounting for the largest portions of the difference (i.e. with the largest amplitude) were established. This approach is a modified version of the discriminant pursuit method [25]. Thus, five coefficients were extracted for each of the 32 EEG channels, totaling in a feature pool of size 160 for classification. The processing was performed using the Matlab wavelet toolbox.

### Classifiers

Two classifiers were investigated in this study: the non-linear artificial neural networks (ANNs) and multiple linear regression (MLR).

An ANN is a biologically inspired information processing paradigm comprised of a network of highly interconnected units called neurons [20]. A feed-forward network is constructed by connecting a number of these neurons in layers. The output of a neuron in layer *j*, out of *m*, is computed as:

yj=σ∑i=1nwijxi,j=1,...,m
 MathType@MTEF@5@5@+=feaafiart1ev1aaatCvAUfKttLearuWrP9MDH5MBPbIqV92AaeXatLxBI9gBaebbnrfifHhDYfgasaacH8akY=wiFfYdH8Gipec8Eeeu0xXdbba9frFj0=OqFfea0dXdd9vqai=hGuQ8kuc9pgc9s8qqaq=dirpe0xb9q8qiLsFr0=vr0=vr0dc8meaabaqaciaacaGaaeqabaqabeGadaaakeaafaqabeqacaaabaGaemyEaK3aaSbaaSqaaiabdQgaQbqabaGccqGH9aqpiiGacqWFdpWCdaaeWbqaaiabdEha3naaBaaaleaacqWGPbqAcqWGQbGAaeqaaOGaemiEaG3aaSbaaSqaaiabdMgaPbqabaaabaGaemyAaKMaeyypa0JaeGymaedabaGaemOBa4ganiabggHiLdGccqGGSaalaeaacqWGQbGAcqGH9aqpcqaIXaqmcqGGSaalcqGGUaGlcqGGUaGlcqGGUaGlcqGGSaalcqWGTbqBaaaaaa@4AF5@

where *n *is the number of units in the preceding layer, *x*_*i *_is the outputs from the preceding layer, and *w*_*ij *_are the corresponding weights. The sigmoidal activation function used here is:

σ(s)=11+e−s
 MathType@MTEF@5@5@+=feaafiart1ev1aaatCvAUfKttLearuWrP9MDH5MBPbIqV92AaeXatLxBI9gBaebbnrfifHhDYfgasaacH8akY=wiFfYdH8Gipec8Eeeu0xXdbba9frFj0=OqFfea0dXdd9vqai=hGuQ8kuc9pgc9s8qqaq=dirpe0xb9q8qiLsFr0=vr0=vr0dc8meaabaqaciaacaGaaeqabaqabeGadaaakeaaiiGacqWFdpWCcqGGOaakcqWGZbWCcqGGPaqkcqGH9aqpdaWcaaqaaiabigdaXaqaaiabigdaXiabgUcaRiabdwgaLnaaCaaaleqabaGaeyOeI0Iaem4Camhaaaaaaaa@394B@

An ANN learns a given task by training, that is, adapting its weights according to given training data. A properly designed and trained network is insensitive to noise and can approximate solutions to problems it has previously not been exposed to. ANNs fully model the task internally and no mathematical parameterization of the problem is required. The ANN topology must, however, be specified, either by optimization methods or empirically.

The MLR model, for a system with *m *observations and *n *features, is typically stated as:

yj=∑i=1nαijxij+βj,j=1,...,m
 MathType@MTEF@5@5@+=feaafiart1ev1aaatCvAUfKttLearuWrP9MDH5MBPbIqV92AaeXatLxBI9gBaebbnrfifHhDYfgasaacH8akY=wiFfYdH8Gipec8Eeeu0xXdbba9frFj0=OqFfea0dXdd9vqai=hGuQ8kuc9pgc9s8qqaq=dirpe0xb9q8qiLsFr0=vr0=vr0dc8meaabaqaciaacaGaaeqabaqabeGadaaakeaafaqabeqacaaabaGaemyEaK3aaSbaaSqaaiabdQgaQbqabaGccqGH9aqpdaaeWbqaaGGaciab=f7aHnaaBaaaleaacqWGPbqAcqWGQbGAaeqaaOGaemiEaG3aaSbaaSqaaiabdMgaPjabdQgaQbqabaGccqGHRaWkcqWFYoGydaWgaaWcbaGaemOAaOgabeaaaeaacqWGPbqAcqGH9aqpcqaIXaqmaeaacqWGUbGBa0GaeyyeIuoakiabcYcaSaqaaiabdQgaQjabg2da9iabigdaXiabcYcaSiabc6caUiabc6caUiabc6caUiabcYcaSiabd2gaTbaaaaa@4EC8@

where, for pattern number *j*, out of *m*, *y*_*j *_is the category estimation, *x*_*ij *_is the *i *: th feature in the feature subset of size *n*, and *α*_*ij *_and *β*_*j *_are parameters that must be established.

### Evolutionary Classifier and Feature Subset Optimization

There are two distinct stages to our classification procedure: classifier parameter approximation and feature subset selection. In the wrapper feature selection approach, the classifier parameters and feature subset are tailored to the given problem simultaneously. In contrast, for filter feature selection the classifier parameters and the feature subsets are optimized separately. In the filter approach, traditional methods (least squares estimation for the MLR and standard back-propagation [20] for the ANNs) are used for establishing the classifier parameters, whereas in the wrapper scheme these (including ANN topology) are determined using evolutionary algorithms (EAs).

An EA is an optimization scheme inspired by Darwinian evolution, where potential problem solutions are encoded as individuals in a population [26]. A fitness measure is computed for each individual, after which various genetic operations, including reproduction, mutation and recombination are applied. A few individuals that will parent the next generation are selected according to a given stochastic scheme, in which probability of selection is related to relative fitness. To ensure that the maximum fitness of the population never decreases, the fittest individual is replaced in the new population unchanged. This process is repeated until performance decreases, evolution stagnates, or a pre-set number of generations have been completed.

In this study, tournament selection is used and the fitness is computed as the proportion of correctly classified epochs. Crossover has been proven to ruin rather than improve the distributed structure of ANNs [18], and has therefore been omitted. Mutation operations are attempted according to given mutation rates, and can be either structural (modifying classifier architecture, i.e. ANN topology) or parametric (modifying classifier parameters, i.e. ANN weights, MLR parameters and feature substitution). The wrapper mutation operations are summarized in tables 1 and 2. ANN hidden neuron addition is performed by adding a neuron with weak, random weights.

**Table 1:** Wrapper ANN mutation operations

Parametric mutation operations
Weight mutation
Structural mutation operations
Feature substitution
Weak hidden node addition
Hidden node removal

**Table 2:** Wrapper MLR mutation operations

Parametric mutation operations
Coefficient mutation
Threshold mutation
Structural mutation operations
Feature substitution

The ANN weights are mutated as follows:

*w *= *ηc *+ *rw*

where *c *is a random number in the range [-3 3] and *r *is a random normally distributed number in the range [0 2]. That is, depending on the random numbers, the classifier parameters are modified drastically or partially. *η *is a mutation step control parameter that initially is set to 1 and adjusted downwards if evolution stagnates.

The ANN and MLR feature substitution operation, utilized for both filter and wrapper optimization, involves substituting a given feature for another, randomly selected from the pool of unused features. Although not done here, it should be pointed out that the number of features can also be subject to optimization for the wrapper as well as the filter approach.

The algorithms were implemented in Matlab and C on a standard PC by one of the authors (M. Åberg), and EEGlab software was used for scalp visualization [27]. For reference, randomly selected feature subsets were evaluated as well.

In summary, the following classification schemes were investigated:

1. **Linear random**: Multiple linear regression with least squares estimation of classifier parameters and a randomly generated feature subset.

2. **Non-linear random**: Artificial neural networks with back-propagation weight estimation and a randomly generated feature subset.

3. **Linear filter**: Multiple linear regression with least squares estimation of classifier parameters and optimization of feature subset.

4. **Non-linear filter**: Artificial neural networks with back-propagation weight estimation and optimization of feature subset.

5. **Linear wrapper**: Multiple linear regression with optimization of classifier parameters and feature subset.

6. **Non-linear wrapper**: Artificial neural networks with optimization of weights, architecture and feature subset.

For the filter methods, 50% of the training data was used for evolutionary fitness computation, and 50% was used for establishing classifier parameters. The fitness was computed on fully trained classifiers. For the wrapper methods, where classifier parameter estimation and feature subset optimization are integrated, only one training dataset is required for evaluating the fitness.

## Results and Discussion

### Results

#### Summary of data

A 32-channel EEG was recorded during self-paced index finger extension for four untrained subjects. The acquired data was pre-processed, and 100 epochs of one second before onset of movement and 0.5 second after were extracted. The data was divided into 80% training and 20% validation data. The discrete wavelet transform was applied the EEG signal and five coefficients for each channel were obtained, resulting in 160 features in total. Classification of the resulting right/left finger movement epochs were attempted using multiple linear regression (MLR) and non-linear artificial neural networks (ANN) with different degrees of evolutionary optimization, including filter and wrapper feature selection.

#### Prediction accuracy

**Figure 1:**
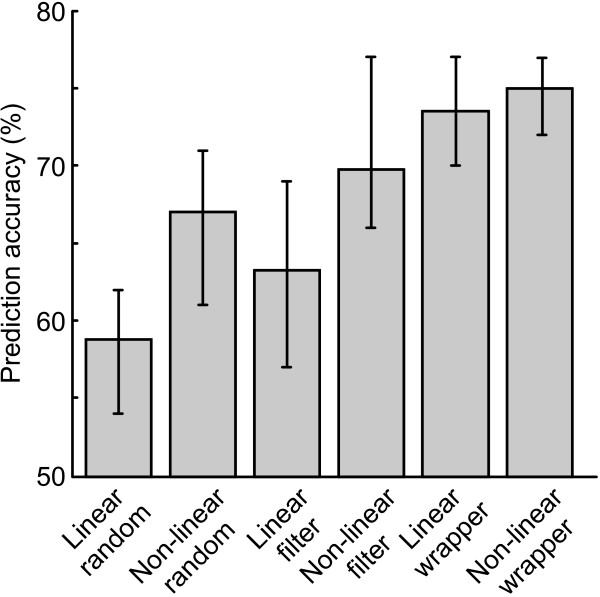
**Classification performance**. Subject mean validation accuracy for the six approaches using only 10 features and 100 patterns. Subject range is indicated by the error bars. The performance appears to increase with increased classifier complexity and tailoring, and the non-linear methods perform better than the linear (*p *< 0.05). The mean difference between wrapper non-linear and wrapper linear is small, suggesting that a high degree of classifier and subset tailoring is more critical than non-linearity. The random feature selection performance is significantly lower than the high-performing wrapper classifiers (*p *< 0.01).

Due to the random nature of evolutionary algorithms and ANN training, each method was run 10 times and the best validation result was obtained. This process was repeated five times, and an average was formed and reported here. In order to compare the different approaches under similar conditions, all methods were restricted to select only 10 out of the 160 features. Random classification results in a score of 50%. The subject mean classification performances appear proportional to classifier complexity (Fig. 1). The non-linear methods performed consistently better than the linear approaches (subject mean for linear random, filter and wrapper: 58.75%, 63.25% and 73.50%, respectively; for non-linear random, filter and wrapper: 67%, 69.75.%, 75%, respectively; *p *= 0.016 using Friedman's non-parametric two-way ANOVA to test for the difference between linear vs. non-linear on subject levels while adjusting for possible method effects [28]). The random feature selection achieved a significantly lower score (linear: 58.75%, non-linear: 67%) than the high-performing wrapper classifiers (linear: 73.50%, non-linear: 75%; Friedman, *p *< 0.01, including adjustment for multiple comparisons), whereas not enough subjects were included to establish any significance of the observed difference between filter (linear: 63.25%, non-linear: 69.75%) and random or wrapper. There was a 7% increase in discrimination success between non-linear filter and non-linear wrapper, whereas the increase between linear filter and linear wrapper was more drastic at 13.95%. There was high variability between subjects in terms of preferred classifier: for subjects 2 and 3, for example, there was little difference between the wrapper and non-linear filter methods. The variability is, however, much lower for the wrapper approach (average range 5%) than the other methods (average range 10.25%).

#### Feature subset selection

**Figure 2:**
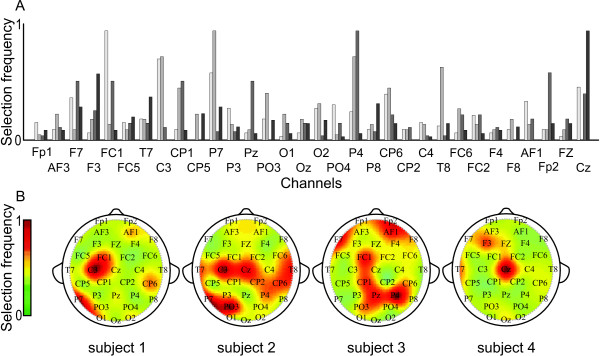
**Feature selection frequency**. Relative frequency of selection for all four subjects per EEG channel (A) and projected on a head model (B). There are clear selection preferences, and although there is high inter-subject variation, FC1, C3 or Cz is highly selected in all subjects. Individual rankings have been scaled to the range [0 1], and the reported results are from the linear filter method.

The constitution of the final feature subsets differs between algorithm runs. However, taking all selected feature subsets into account, a selection frequency ranking is obtained. When the ranking is plotted per EEG channel, it is clear that some electrodes discriminate finger movements better than others (Fig. 2). There is large variation in spatial preference between individuals, yet one pattern is discernible: either of FC1, C3 or Cz is highly selected in all subjects. Plotting the frequency of selection against the wavelet coefficients, on the other hand, did not reveal any time or scale preference.

### Discussion

This study has demonstrated that individual classifier tailoring and feature subset selection significantly improves single-trial limb laterality discrimination, and that the optimal EEG channels differ much between subjects.

It should be noted that this study has focused on comparing classifiers rather than maximizing prediction accuracies. The number of features as well as the maximum generations allowed in the evolutionary algorithm were heavily limited due to time and computer restrictions. Similarly, the number of included movement epochs was reduced to only 100, a factor that significantly decreases the prediction accuracy. The non-linear classifiers performed better than the linear approaches, agreeing with previous studies [29,30]. Interestingly, the improvement between linear and non-linear classifiers is 14.04%, 10.28% and 2.04%, respectively, for the random, filter and wrapper approaches. This observation suggests that as the association between classifier training and feature selection increases, the non-linearity of the classifier becomes less important. In the evolutionary approach, the tailored feature subset is allowed to express either non-linearities or linearities in the data – whatever suits the given classifier optimally. More data is, however, required to establish this theory statistically.

The spatial preference found in this study partially agrees with previous research, which focuses on a few central electrodes for finger movement classification [4-8]. Channels FC1, C3 or Cz, highly selected in all subjects, are located close to the left-hand side motor cortex. Interestingly, C4, the right-hand side equivalent, is not ranked high in any subject, including the left-handed subject 3. Also, in three out of four subjects, P7 or P4 in the parietal regions, with no major established connection to motor areas, are ranked highly. However, the results can in part be explained by investigating the geometrical orientation of the electric fields (dipoles) generated by the activated neurons. For example, projecting the event-related potential for subject 3 on a human head model reveals that the signal source at peak EEG activity before the movement, -39 ms, has a tangential orientation. These results are in accordance with the physiological orientation of the pyramidal neurons responsible for finger movement. The neurons are located in the finger area of the motor cortex, which in turn is located within the central sulcus [9,31]. These pyramidal neurons are aligned along the surface of the cortex, thus generating a tangential dipole [1]. During the course of movement, different areas with different dipole orientations are activated, and it is therefore difficult to manually predict what combination of electrode locations will provide most useful information for laterality discrimination.

Different areas of the cortex are likely to be of varying importance at different times throughout a movement epoch, and each wavelet coefficient corresponds to a given scale – translatable into frequency – and point in time. In this study, however, no single wavelet coefficient was significantly more frequently selected than any other, indicating either that there was no time or frequency preference or that these wavelet coefficients capture the dynamics during finger extension cycle poorly. Not restricting the feature pool to a given number of wavelet coefficients based on an average, as was done here, could potentially resolve this issue.

Ideally, the feature pool would consist of several different types of signal parameters other than wavelet coefficients, such as Fourier frequencies, power spectral densities and autoregressive coefficients. Other state-of-the-art classifiers, such as support vector machines, can also be incorporated into the algorithm.

## Conclusion

The evolutionary design was successful in optimizing classifier parameters and structure, including input features. Higher degrees of tailoring resulted in increased classification accuracies, and non-linear classifiers achieved better results than linear. There was high variation between the resulting features selected for each subject, indicating that a systematic method for accommodating individual variability is useful for single-trial EEG analysis.

## Competing interests

The author(s) declare that they have no competing interests.

## Authors' contributions

MÅ carried out algorithm implementation and evaluation and drafted the manuscript. JW conceived of the study, and participated in its coordination. Both authors participated in study design and data acquisition and read and approved the final manuscript.
